# Bio-Inspired Swarm Optimization for Interpretable Classification of *Tropinota hirta* Abundance Using Partial SMOTE and XGBoost

**DOI:** 10.3390/biomimetics11100738

**Published:** 2026-10-14

**Authors:** Cebrail Barut, İnanç Özgen

**Affiliations:** 1 Department of Continuing Education Center, Firat University, 23119 Elazig, Turkey; cbarut@firat.edu.tr; 2 Department of Bioengineering, Firat University, 23119 Elazig, Turkey

**Keywords:** *Tropinota hirta*, almond orchard, blue basin trap, trap capture, pest abundance classification, XGBoost, Particle Swarm Optimization, Partial SMOTE, class imbalance, interpretable machine learning

## Abstract

Classifying blue basin trap captures of *Tropinota hirta* according to environmental conditions can support timely and data-driven management of this flower-feeding pest in almond orchards; however, imbalanced class distributions pose important challenges for machine-learning models. This study proposes an interpretable PSO-XGBoost with Partial SMOTE framework for classifying *T. hirta* abundance levels derived from blue basin trap captures using temporal and meteorological variables. From a biomimetic perspective, Particle Swarm Optimization (PSO) is a bio-inspired optimization algorithm that emulates the collective search and information-sharing behavior observed in natural swarms, translating principles of biological collective intelligence into a computational optimization process. The dataset consisted of 256 trap observations categorized into High (H), Low (L), and Medium (M) abundance classes, with 180, 55, and 21 observations, respectively, indicating substantial class imbalance. Eleven temporal and meteorological variables were used as predictors, while PestCount, representing the observed number of *T. hirta* adults captured in the blue traps and used to derive the target classes, was excluded from the predictor set to prevent target leakage. The proposed framework combines controlled minority-class oversampling using Partial SMOTE, bio-inspired PSO for XGBoost hyperparameter optimization, and rule extraction for model interpretability. A Partial-SMOTE ratio ablation analysis identified L = 0.80 and M = 0.40 as the most favorable class-specific oversampling configuration, avoiding complete class balancing while improving overall and class-sensitive predictive performance. Performance was evaluated using stratified five-fold cross-validation, with oversampling applied exclusively to the training folds to prevent information leakage. The proposed method achieved a mean Accuracy of 89.45% and a mean Weighted F1-score of 88.79%, representing the best overall performance among the evaluated methods. The ablation analysis showed that PSO-based optimization improved conventional XGBoost, while the proposed Partial-SMOTE configuration outperformed both class-weighted XGBoost and PSO-XGBoost without oversampling. Robustness was further assessed using 10-times repeated stratified five-fold cross-validation, where the proposed method achieved 87.51 ± 3.75% Accuracy, 86.94 ± 3.71% Weighted F1-score, and 71.60 ± 7.40% Macro F1-score. Friedman and post-hoc Wilcoxon signed-rank tests with Holm correction confirmed statistically significant performance differences in favor of the proposed method. In addition, human-readable IF–THEN rules with bounded temporal and meteorological conditions were extracted from the optimized model to improve interpretability. The results indicate that integrating a biomimetic swarm-intelligence optimization strategy with controlled oversampling, blue-trap monitoring, and interpretable machine learning provides a promising framework for predicting *T. hirta* abundance levels and supporting environmentally compatible monitoring and pest-management decision-making in almond orchards.

## 1. Introduction

Almond (*Prunus dulcis* [Mill.] D.A. Webb) is an economically important fruit species, particularly in arid and semi-arid regions. Successful almond production depends strongly on the protection of floral organs and adequate pollination during the relatively short flowering period. Pest activity during this stage can damage sensitive flower tissues, interfere with fertilization, and reduce fruit set and yield. Among the pests associated with flowering fruit trees, *T. hirta* (Poda, 1761) (Coleoptera: Scarabaeidae: Cetoniinae) is a polyphagous flower-feeding species of agricultural importance. *T. hirta* has also been reported at substantial population densities in almond orchards in Türkiye, where blue basin traps have been used to monitor adult activity during the growing season [1]. Adults may feed on petals, stamens, pistils, pollen, and other floral tissues of several fruit species, including almond, apple, pear, apricot, peach, and cherry [2]. Its economic importance is particularly associated with the coincidence of adult flight activity with budding and flowering periods. Intensive feeding during this stage may impair fertilization and reduce fruit set, while unfavorable environmental conditions such as rainfall, low temperature, or frost can further increase production risks.

Effective management of *T. hirta* is challenging because its activity period overlaps with flowering, when honey bees and other pollinators are active in orchards. For this reason, monitoring-based and environmentally compatible management strategies are particularly important. Visual traps, especially blue traps, have been used to monitor adult emergence and population intensity [2,3]. Trap captures and adult activity may also vary temporally and in relation to environmental conditions such as temperature [3]. The effectiveness of visual trapping can also be enhanced by chemical attractants, particularly combinations of cinnamyl alcohol and trans-anethole, which have been proposed for *T. hirta* monitoring [4].

Because pest abundance changes over time in response to environmental conditions, fixed-calendar management strategies may be insufficient for identifying periods of elevated pest activity. Integrating pest observations with meteorological and temporal variables can provide a more dynamic basis for pest-abundance prediction, monitoring, and pest-management decision support. Recent studies have also demonstrated the potential of machine-learning approaches for forecasting insect abundance from monitoring time series, supporting more timely pest-management decisions [5]. In this context, machine learning provides an opportunity to model nonlinear relationships between environmental conditions and pest abundance levels. Nevertheless, developing reliable classification models from field-based pest monitoring data remains challenging. Such datasets may contain relatively few observations and highly imbalanced target classes, while advanced ensemble models can also be sensitive to hyperparameter configuration [6]. In addition, highly predictive models may provide limited information about the environmental conditions underlying their decisions [7]. Therefore, class imbalance, model optimization, predictive performance, and interpretability need to be considered jointly.

From a biomimetics perspective, swarm intelligence provides a computational paradigm inspired by the collective behavior and self-organization observed in biological systems. Particle Swarm Optimization (PSO), originally inspired by the coordinated movement of natural swarms such as bird flocks and fish schools, translates principles of individual exploration, social interaction, and collective adaptation into a population-based optimization process [8]. In PSO, candidate solutions interact through shared information about individual and collective search experience, enabling the population to progressively explore and exploit the solution space. In the present study, this bio-inspired mechanism is employed to optimize the hyperparameters of XGBoost. Thus, the biomimetic component of the proposed framework lies in transferring principles of natural swarm intelligence to the computational optimization of a machine-learning model for pest abundance classification.

Accordingly, this study aims to classify blue basin trap captures of *T. hirta* into High (H), Low (L), and Medium (M) abundance levels using temporal and meteorological variables and to develop an accurate and interpretable classification framework for imbalanced trap-monitoring data. The proposed approach integrates Partial SMOTE, bio-inspired Particle Swarm Optimization (PSO), XGBoost, and rule extraction within a unified framework. From a biomimetic perspective, PSO transfers principles of collective search, information sharing, and adaptive behavior observed in natural swarms to the optimization of XGBoost hyperparameters. In this way, the proposed framework combines a nature-inspired optimization mechanism with practical blue-trap monitoring and data-driven pest-abundance prediction and decision support.

The main contributions of the proposed framework are:

- A class-specific controlled Partial SMOTE strategy is employed to improve minority-class representation without forcing complete class balance.
- Class-specific oversampling ratios are used to limit excessive synthetic sample generation and preserve part of the original class structure.
- PSO-based XGBoost hyperparameter optimization is integrated with controlled oversampling while jointly considering Accuracy and Weighted F1-score.
- Bounded IF–THEN rules are extracted from the optimized model to provide human-readable descriptions of the temporal and meteorological conditions associated with different abundance levels.
- The main contribution of the study lies in the problem-specific integration of blue-trap monitoring, meteorological variables, controlled class-imbalance handling, bio-inspired PSO-based optimization, and interpretable rule extraction within a unified framework for *T. hirta* abundance classification.

## 2. Related Works

Machine learning and artificial intelligence techniques have become increasingly important in agricultural pest management, particularly for pest detection, population monitoring, abundance prediction, and decision-support applications. The availability of meteorological, temporal, sensor-based, and field observation data has enabled researchers to investigate complex relationships between environmental conditions and pest population dynamics using data-driven approaches. Various machine learning methods, ranging from conventional classifiers to ensemble and boosting algorithms, have been employed to improve the accuracy and reliability of pest-related predictions. However, field-based agricultural datasets frequently present challenges such as limited sample size, imbalanced class distributions, complex nonlinear relationships, and insufficient model interpretability [7]. Consequently, recent research has increasingly focused on not only improving predictive performance but also addressing class imbalance, optimizing model parameters, and providing more interpretable decision-support models. These research directions are particularly relevant to pest abundance classification, where reliable prediction requires models that can handle limited and imbalanced field observations while maintaining sufficient interpretability for practical decision support.

Wadhwa et al. [9] developed a generalizable and interpretable machine learning framework for the early prediction of rice pests using environmental variables collected from different regions of India. The study considered Green Leafhopper, Yellow Stem Borer, and No Pest as three target classes and incorporated meteorological variables such as temperature, humidity, rainfall, wind speed, sunshine duration, and evaporation. Twelve machine learning algorithms, including Random Forest, Balanced Random Forest, XGBoost, CatBoost, SVM, and LightGBM, were initially evaluated. To address the imbalanced class distribution, in which the No Pest class represented 62% of the observations, the authors employed SMOTE-ENN, combining synthetic minority oversampling with the removal of noisy and ambiguous samples. The selected models were subsequently optimized using Optuna, and the tuned CatBoost model achieved an overall F1-score of 0.9414 and a mean AUC of 0.9912. Furthermore, SHAP analysis was employed to explain the contribution of environmental variables to pest occurrence, demonstrating the potential of combining imbalance handling, hyperparameter optimization, and explainability in environmental data-driven pest forecasting.

Wang et al. [10] proposed an explainable deep learning framework for forecasting pest outbreaks from meteorological and historical pest data. Their Attention-based Long Short-Term Memory Interaction Convolutional Neural Network (ALIC) combines a feature-attention mechanism, LSTM networks for capturing temporal dependencies, and an Interaction Convolutional Neural Network for modeling interactions between meteorological and pest-related variables. The framework was evaluated using real-world observation data from Huiyang and Shantou in Guangdong Province, China, and outperformed the benchmark models across different forecasting horizons. Feature-importance analysis further indicated that temperature and rainfall were among the most influential variables affecting pest outbreaks. The study highlights both the importance of meteorological information in pest forecasting and the need for interpretable models capable of revealing the environmental factors underlying predictions.

Rodríguez-Díaz et al. [11] investigated explainable machine learning approaches for forecasting olive fly and red spider mite populations in olive groves and vineyards. Four datasets containing information on crop phenology, pest populations, damage levels, and meteorological conditions were analyzed using classification and regression models implemented through the H2O framework. In particular, the classification problem included the prediction of intervals corresponding to the number of olive flies captured in traps, making the study relevant to pest abundance level prediction. Explainability was incorporated through SHAP values, feature importance, and Individual Conditional Expectation plots. The analysis identified solar radiation and wind direction as important factors for olive fly predictions, whereas previous pest occurrence and wind velocity were influential for red spider mite forecasting. These findings demonstrate that meteorological variables can provide meaningful information for both pest-level prediction and interpretable agricultural decision support.

Arora et al. [12] investigated the population dynamics of *Frankliniella occidentalis* under two contrasting agricultural microclimates using machine learning. The study analyzed 1686 weekly trap observations, standardized into 2254 modeling units, together with 16 environmental predictors, and compared Random Forest, Gradient Boosting Machine, and XGBoost models. Random Forest achieved the highest predictive accuracy in open-field conditions at 87.7%, whereas XGBoost provided the best performance in high-tunnel conditions with an accuracy of 84.9%. Parent pest population and temperature consistently emerged as the most influential predictors, while humidity and wind had secondary effects. Importantly, models trained in one microclimate achieved no more than 44% accuracy when transferred to the other environment, emphasizing that pest population dynamics can be strongly dependent on local environmental conditions.

Güral [13] comparatively evaluated machine learning models for predicting the population density of *Cimbex quadrimaculata* using meteorological and vegetation variables collected in Diyarbakır and Elazığ, Türkiye, between 2020 and 2022. The study formulated pest population prediction as three different problems—binary classification, multiclass classification, and regression—and evaluated a broad range of machine learning algorithms. Ensemble boosting methods, particularly Gradient Boosting, XGBoost, and LightGBM, demonstrated strong performance in capturing nonlinear relationships between environmental factors and pest population density. Gradient Boosting achieved 94.3% accuracy and an AUC of 0.983 in the binary classification setting, while XGBoost achieved 84.6% accuracy in the reported classification analyses. SHAP-based interpretation further showed that temperature- and humidity-related variables were consistently among the most influential predictors. This study provides particularly relevant evidence that meteorological information and ensemble learning can be effectively combined for multiclass pest population modeling.

Bal et al. [14] examined the influence of class imbalance and SMOTE-based oversampling on machine learning classification performance in agricultural datasets. The authors used an imbalanced dataset comprising seven date palm and two pistachio cultivars and compared seven machine learning algorithms, including Decision Tree, Random Forest, Logistic Regression, SVM, MLP, KNN, and Naive Bayes. On the original imbalanced dataset, Linear-SVM obtained the highest accuracy of 92.62%, whereas after applying SMOTE, RBF-SVM achieved an accuracy of 95.55%. The study demonstrated that synthetic oversampling can improve classification performance when agricultural datasets contain unequal class distributions. However, their SMOTE strategy aimed to equalize minority classes with the majority class, representing a full balancing approach rather than a controlled partial oversampling strategy.

Longo et al. [15] addressed the problem of highly imbalanced datasets in pest detection and classification. Using the IP102 dataset, which contains more than 75,000 images distributed across 102 pest categories, the authors emphasized that natural pest distributions can lead machine learning models to favor highly represented classes while disregarding minority classes. Rather than relying on conventional oversampling or undersampling alone, they proposed a multi-stage balancing procedure in which overrepresented classes are divided into correlated subclasses without deleting original observations. The authors also highlighted an important limitation of synthetic oversampling: excessive generation of synthetic observations may add little new information and potentially increase overfitting. Their findings reinforce the importance of carefully controlling the degree of class balancing in pest classification problems rather than assuming that complete class equalization is always optimal.

Beyond agricultural applications, SMOTE has also been combined with XGBoost for imbalanced classification problems. Han et al. [16] compared Borderline-SMOTE, random downsampling, random oversampling, conventional SMOTE, and ADASYN, and subsequently integrated the selected sampling strategy with XGBoost. Their findings further demonstrate the practical compatibility of synthetic oversampling and boosting-based classification for imbalanced datasets.

From a biomimetics perspective, swarm intelligence provides a computational approach in which principles observed in the collective behavior of biological systems are transferred to optimization problems. Particle Swarm Optimization (PSO), originally inspired by coordinated behaviors observed in natural swarms, represents this concept by allowing candidate solutions to adapt their search trajectories according to both individual experience and information shared by the swarm [1]. Through this interaction, PSO translates biological principles of collective search, information sharing, and adaptive behavior into a computational optimization mechanism. This nature-inspired characteristic has encouraged the integration of PSO with machine-learning models in agricultural applications, particularly for feature selection and hyperparameter optimization.

Yu et al. [17] proposed a PSO-XGBoost framework for estimating daily reference evapotranspiration in solar greenhouse environments using meteorological and soil-moisture variables. In this framework, XGBoost served as the predictive model, while Particle Swarm Optimization was employed to optimize six important hyperparameters, including learning rate, maximum depth, minimum child weight, gamma, subsample, and column subsampling ratio. The authors reported that PSO could stably optimize XGBoost parameters and that PSO-XGBoost provided the best evapotranspiration estimation performance compared with the other evaluated ensemble learning approaches. Although the study addressed an agricultural regression problem rather than pest abundance classification, it provides methodological evidence for the effectiveness of PSO-based XGBoost hyperparameter optimization when working with meteorological agricultural data.

Ding et al. [18] proposed a hybrid GA-PSO-XGBoost framework for classifying potato yield into low, medium, and high levels using agricultural sensor data collected between 2015 and 2023. The model used an 18-dimensional feature set containing phenological, hydrothermal, and soil-related variables. PSO was used as the main optimization mechanism, while genetic crossover and mutation operations were periodically introduced to improve population diversity and reduce the risk of convergence to local optima. Using five-fold cross-validated Macro F1 as the optimization objective, the proposed GA-PSO-XGBoost model achieved a Macro F1-score of 0.872 ± 0.01, compared with 0.788 ± 0.02 for conventional XGBoost and 0.863 ± 0.01 for PSO-XGBoost. The model also achieved an accuracy of 0.861. This study demonstrates the effectiveness of metaheuristic optimization for improving XGBoost performance in imbalanced multiclass agricultural classification problems.

Subaşi [19] investigated metaheuristic hyperparameter optimization for XGBoost in a seven-class dry bean classification problem. Critical XGBoost parameters, including learning rate, tree depth, and subsampling ratios, were optimized using several algorithms, including Particle Swarm Optimization, Grey Wolf Optimizer, Slime Mould Algorithm, modified SMA, and the proposed mSMA_plus approach. These methods were also compared with conventional GridSearch and RandomSearch optimization. The proposed mSMA_plus-XGBoost model achieved the highest reported classification accuracy of 99.39% and an F1-score of 0.9939. Although PSO was not the best-performing optimizer in this study, the comparative results demonstrate the importance of metaheuristic hyperparameter search for XGBoost and support its application in complex multiclass agricultural classification tasks.

Thihlum et al. [20] proposed an explainable XGBoost-based crop recommendation framework integrating Random Forest, Particle Swarm Optimization, SMOTE, and SHAP. The study first employed Random Forest to identify important agricultural features and subsequently used PSO to select an optimized subset of these variables. SMOTE was applied to address the class imbalance problem before training the XGBoost classifier, while five-fold cross-validation was used to evaluate generalization performance. The study is particularly relevant because it integrates feature optimization, imbalance handling, XGBoost classification, and model explainability within a single agricultural machine learning framework. However, unlike the present study, PSO was primarily employed for feature subset optimization rather than direct XGBoost hyperparameter optimization, and interpretability was provided through SHAP rather than explicit IF–THEN decision rules.

Venkateswara and Padmanaban [8] proposed an interpretable deep-learning framework for agricultural crop and fertilizer recommendation using TabNet and SHAP. The study combined SMOTE-based class balancing with five-fold cross-validation and employed SHAP to explain the contribution of agricultural and environmental variables to model predictions. Their findings highlight the increasing importance of combining predictive performance, imbalance handling, and model interpretability in agricultural decision-support systems.

Coulibaly et al. [21] investigated explainable deep convolutional neural networks for insect pest recognition using more than 75,000 images belonging to 102 pest categories in the IP102 dataset. Their approach combined CNN-based pest identification with several explainability techniques to visualize and localize image regions that contributed to model predictions. By combining explanation maps and evaluating their relationships using mutual information, the authors aimed to make CNN decisions more understandable to end users. The study also reported a 58.90% reduction in the number of network parameters while maintaining an interpretable pest-recognition framework. Although this approach focuses on image-based pest recognition rather than meteorological pest abundance classification, it demonstrates the growing importance of transparency and human-understandable explanations in AI-supported pest management systems.

Barut et al. [22] proposed an explainable hybrid Random Forest–Ant Colony Optimization (RF-ACO) model for classifying five cherry fruit coloration stages using pomological characteristics and cherry fruit fly density data. In the proposed framework, Random Forest was used to generate a large pool of candidate decision rules, while Ant Colony Optimization was employed to select a compact and highly discriminative subset of rules. The extracted decision paths were converted into interval-based IF–THEN expressions with explicit lower and upper bounds, enabling experts to interpret the model’s decision logic directly. The proposed RF-ACO model achieved an accuracy of 99.48%, outperforming 14 conventional machine learning methods. Feature importance analysis identified soluble solids content, cherry fly density, NaOH, and acidity as the most influential variables. This study is particularly relevant to the present work because it demonstrates how tree-based ensemble models can be transformed into transparent, bounded decision rules while preserving high predictive performance.

Metaheuristic optimization has also been increasingly investigated to improve the performance of XGBoost-based agricultural prediction models. Yu et al. proposed a PSO-XGBoost model for estimating reference evapotranspiration in solar greenhouses, where PSO was employed to optimize XGBoost parameters using meteorological and soil-related variables, and the optimized model demonstrated improved estimation performance compared with alternative ensemble approaches. More recently, metaheuristic optimization has been extended to multiclass agricultural classification problems. A GA-PSO-XGBoost framework was developed for classifying potato yield levels using phenological, hydrothermal, and soil variables, demonstrating the potential of swarm-based optimization for improving both classification accuracy and Macro F1-score. Similarly, recent research on multiclass dry bean classification compared several metaheuristic strategies, including PSO, for optimizing critical XGBoost hyperparameters and reported strong classification performance for the optimized models. Kumar et al. further combined XGBoost, PSO-based feature selection, SMOTE, and SHAP-based interpretability for crop recommendation, highlighting the potential of integrating optimization, imbalance handling, and explainability within agricultural machine learning systems. Recent studies have also introduced new optimization algorithms for complex global and stochastic optimization problems, including the Secant Optimization Algorithm [23] and the Schrödinger Optimizer [24]. These approaches illustrate the continuing development of alternative metaheuristic and physics-inspired optimization strategies beyond established methods such as PSO. Although the present study focuses on PSO-based hyperparameter optimization, the evaluation of such recent optimizers within the proposed framework may provide a useful direction for future comparative research. Nevertheless, these studies primarily focus on evapotranspiration estimation, crop recommendation, crop yield, or crop-type classification rather than pest abundance classification under severe class imbalance. Moreover, although PSO has been widely employed as an optimization technique, its role as a biomimetic mechanism that transfers principles of natural swarm intelligence to machine-learning optimization has received comparatively limited emphasis in pest abundance modeling. This distinction motivates the development of the present framework, which combines controlled minority oversampling, bio-inspired PSO-based XGBoost hyperparameter optimization, and interpretable rule extraction for pest abundance classification.

## 3. Materials and Methods

### 3.1. Dataset and Study Variables

The dataset used in this study consists of 256 valid observations obtained after the data preprocessing stage. Each observation represents a blue basin trap record for adult *T. hirta* associated with the corresponding temporal and meteorological conditions. The prediction problem was formulated as a three-class classification task. The dataset includes temporal and meteorological variables together with the observed blue-trap catch, recorded as PestCount.

The categorical target variable (Class) was derived from PestCount, namely the number of *T. hirta* adults captured in the blue basin traps. Observations with a PestCount between 0 and 14 were assigned to the Low (L) class, observations between 15 and 29 were assigned to the Medium (M) class, and observations with a PestCount of 30 or greater were assigned to the High (H) class. Following this categorization, the final dataset contained 180 observations belonging to class H, 55 observations belonging to class L, and 21 observations belonging to class M, indicating a considerable class imbalance. These thresholds were defined in a data-driven manner by considering the distribution of pest-count observations in the dataset. They were used as study-specific operational abundance categories to distinguish different levels of pest activity for the classification task. Therefore, these cut-off values should not be interpreted as universal economic injury or pest-control intervention thresholds.

Eleven predictor variables were used as input features: Year, Month, DayOfYear, Max. Temperature, Min. Temperature, Mean Temperature, Humidity, Precipitation, Wind Speed, Wind Angle, and Atmospheric Pressure. These variables represent temporal and meteorological conditions potentially associated with changes in pest abundance. The inclusion of Year provides an additional temporal variable that distinguishes observations collected across different years, while Month and DayOfYear represent seasonal and within-year temporal variation.

Although PestCount was retained in the dataset as the original numerical blue-trap catch from which the target classes were generated, it was not included as an input feature in any classification model. Since the target variable Class was directly derived from the number of *T. hirta* adults captured, including PestCount among the predictors would introduce direct target leakage and could lead to artificially inflated classification performance. Therefore, all machine learning models were trained exclusively using the eleven temporal and meteorological predictor variables.

After preprocessing, the resulting feature matrix consisted of 256 observations and 11 predictor variables, while the target variable consisted of the three classes H, L, and M. A summary of the variables included in the dataset, together with their data types, roles in the classification process, and descriptions, is presented in Table 1.

### 3.2. Data Preprocessing and Experimental Setup

Prior to model development, the dataset was subjected to a preprocessing procedure to ensure data consistency and prevent information leakage. Completely empty and irrelevant columns were removed, and the selected predictor variables were converted into numerical form where necessary. Observations containing missing values in the predictor variables or the target variable were excluded from the analysis. Following preprocessing, 256 valid observations with 11 predictor variables remained for model development and evaluation.

The PestCount variable was excluded from the predictor set because the target variable, Class, was directly derived from this variable, as described in Section 3.1. Including PestCount as a predictor would therefore introduce target leakage and could result in an artificially high estimate of model performance. Consequently, only the temporal and meteorological variables listed in Table 1 were used as model inputs. The categorical target labels H, L, and M were numerically encoded to ensure compatibility with the classification algorithms. The same predictor set and target definition were maintained across all experiments to provide a consistent basis for model comparison.

Due to the relatively small sample size and the imbalanced distribution of the target classes, model performance was evaluated using stratified five-fold cross-validation. The dataset was divided into five folds while preserving the original class proportions as closely as possible. In each iteration, four folds were used for model training and the remaining fold was used for validation. This procedure was repeated until each fold had served once as the validation set. The data were shuffled before partitioning, and a fixed random seed of 42 was used to improve the reproducibility of the experiments. For experiments involving oversampling, the resampling procedure was applied exclusively to the training portion of each cross-validation fold, while the validation fold was retained in its original form. Thus, synthetic samples were generated only from the training observations, preventing information from the validation data from influencing the training process and reducing the risk of data leakage.

All compared methods were evaluated under the same stratified five-fold cross-validation framework. Accuracy and Weighted F1-score were used as the primary evaluation metrics. Accuracy measures the overall proportion of correctly classified observations, whereas the Weighted F1-score combines class-specific precision and recall while accounting for the number of observations in each class. For each method, the mean performance across the five validation folds was calculated, and the variability across folds was reported using the standard deviation. This evaluation framework provided a consistent basis for comparing the predictive performance of the proposed method with the baseline classifiers.

### 3.3. Proposed PSO-XGBoost with Partial SMOTE Method

**Figure 1:**
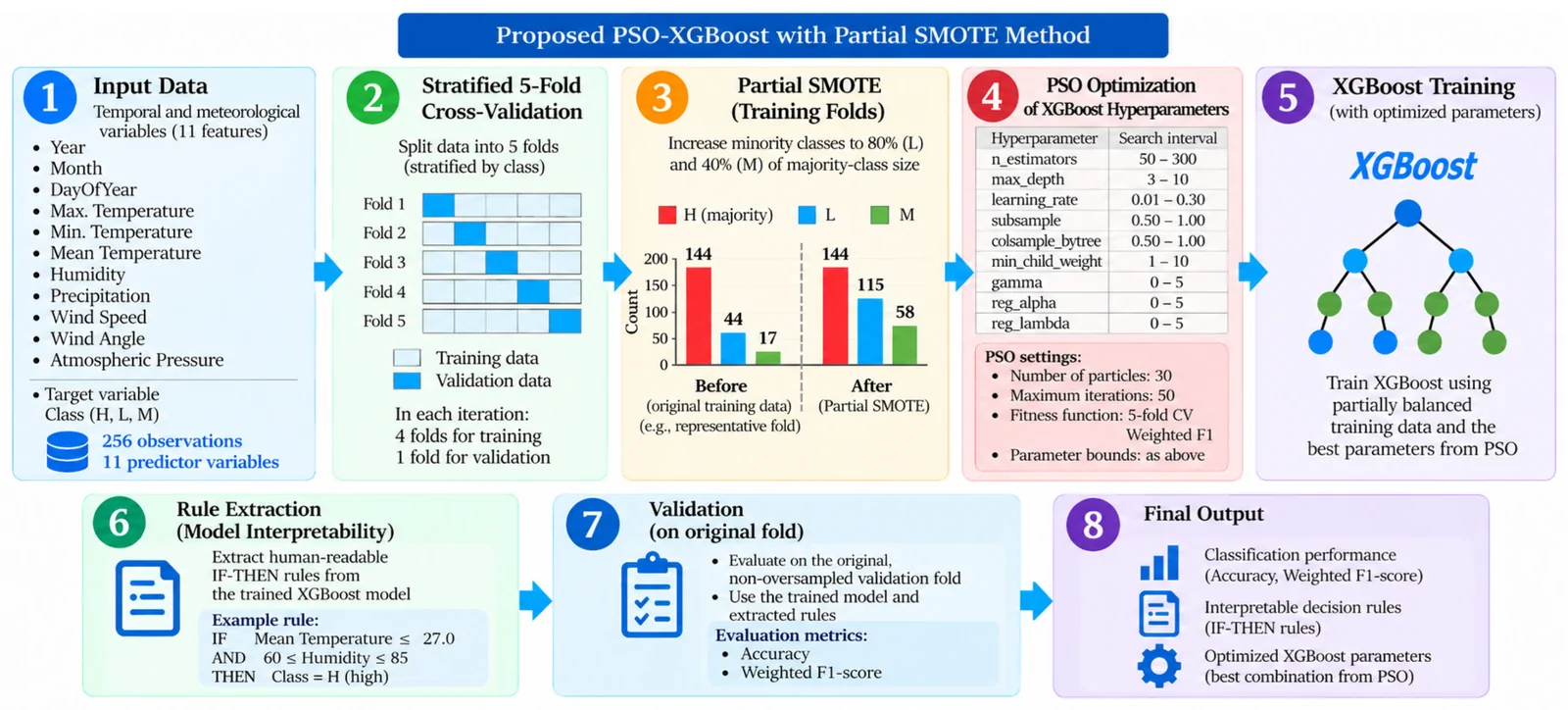
Flowchart of the proposed PSO-XGBoost with Partial SMOTE method.

This study proposes an integrated classification approach combining Partial Synthetic Minority Oversampling Technique (Partial SMOTE), Particle Swarm Optimization (PSO), and Extreme Gradient Boosting (XGBoost) to address both class imbalance and model parameter optimization. The proposed method, referred to as PSO-XGBoost with Partial SMOTE, was designed to improve the classification of pest abundance levels while maintaining high overall predictive performance. The overall workflow of the proposed method is illustrated in Figure 1.

As shown in Figure 1, the proposed approach consists of a sequence of interconnected stages. First, the temporal and meteorological predictor variables are organized using stratified five-fold cross-validation. Partial SMOTE is then applied exclusively to the training folds to reduce the imbalance among the High (H), Low (L), and Medium (M) classes without completely equalizing their sample sizes. Next, PSO is employed to search for an effective combination of XGBoost hyperparameters, and the XGBoost classifier configured with the optimized parameters is trained using the partially balanced training data. The trained model is subsequently evaluated on the original, non-oversampled validation fold using Accuracy and Weighted F1-score. Finally, human-readable IF–THEN rules are extracted from the optimized XGBoost model to improve model interpretability.

The proposed method was implemented within a stratified five-fold cross-validation framework. In each fold, the training and validation subsets were separated before any oversampling operation. Partial SMOTE was applied exclusively to the training subset, whereas the validation subset was preserved with its original class distribution. Consequently, synthetic observations generated during oversampling were never introduced into the validation data, thereby preventing information leakage during model evaluation.

Let $N_{max}$ represent the number of observations in the majority class. The target sample sizes for classes L and M were determined according to Equations (1) and (2), respectively.

(1) $$N_{L}^{Target}=round(0.80\times N_{max})$$

(2) $$N_{M}^{Target}=round(0.40\times N_{max})$$

As defined in Equations (1) and (2), the target sample sizes for classes L and M were set to approximately 80% and 40% of the majority-class size, respectively, while the majority class remained unchanged. For example, a representative training-fold distribution of approximately 144 H, 44 L, and 17 M observations was transformed into approximately 144 H, 86 L, and 65 M observations, rather than completely balancing all three classes. This controlled oversampling strategy reduces the number of synthetic observations while improving the representation of the minority classes.

Synthetic samples are generated using SMOTE with k = 2 nearest neighbors [25]. Because class M contains a limited number of original observations, the effective number of neighbors is dynamically restricted according to the number of available minority-class samples in each training fold. This ensures that the oversampling procedure remains feasible even when only a few minority samples are available.

Within the proposed framework, PSO constitutes the biomimetic optimization component. Inspired by the collective search behavior of natural swarms, PSO represents candidate solutions as particles that adapt their positions in the search space according to their own previous experience and information shared by the swarm [1]. This interaction provides a computational analogue of individual exploration and collective adaptation, enabling the population of candidate solutions to progressively search for effective parameter configurations. In the present study, this bio-inspired search mechanism was used specifically for XGBoost hyperparameter optimization rather than for modeling the biological behavior of *T. hirta*.

Following Partial SMOTE, PSO is used to optimize six XGBoost [26] hyperparameters: the number of estimators (n_estimators), maximum tree depth (max_depth), learning rate (learning_rate), subsampling ratio (subsample), feature subsampling ratio (colsample_bytree), and minimum loss reduction (gamma). The search intervals were 50–300, 2–8, 0.01–0.30, 0.50–1.00, 0.50–1.00, and 0–5, respectively. The PSO procedure used 12 particles and a maximum of 40 iterations. Since the dataset is imbalanced, model optimization was not based solely on Accuracy, as accuracy-based evaluation may provide an incomplete assessment of classifier performance under imbalanced class distributions [27]. Instead, the PSO fitness function was defined as an equally weighted combination of Accuracy and Weighted F1-score

For each candidate PSO solution, the fitness function defined in Equation (3) was used to calculate the fitness value based on the mean Accuracy and mean Weighted F1-score obtained through stratified five-fold cross-validation. An early-stopping mechanism was also used, and optimization was terminated when no improvement greater than 10^−4^ was observed for eight consecutive iterations. The best parameter combination identified by PSO was then used for the final XGBoost evaluation with Partial SMOTE.

(3) Fitness=0.50×Accuracy+0.50×Weighted F1

To improve model interpretability, human-readable IF–THEN rules were extracted from the tree structures of the optimized XGBoost model. Each root-to-leaf decision path was represented as a conjunction of feature-based conditions. When multiple split conditions involved the same predictor, these conditions were combined to form bounded intervals defined by lower and upper limits. The resulting rules therefore describe the temporal and meteorological conditions associated with the predicted abundance classes in a compact and interpretable form. Rule-level accuracy was calculated as the proportion of observations satisfying a rule that belonged to the class predicted by that rule. Because the complete XGBoost ensemble can generate a large number of decision paths, representative rules were selected for interpretation, with three rules reported for each abundance class (H, L, and M).

The overall workflow of the proposed method can therefore be summarized as input data, stratified five-fold cross-validation, Partial SMOTE on the training fold, PSO-based XGBoost hyperparameter optimization, XGBoost training, rule extraction for model interpretation, validation on the original non-oversampled fold, and performance evaluation.

## 4. Results

### 4.1. Comparative Classification Performance

**Figure 2:**
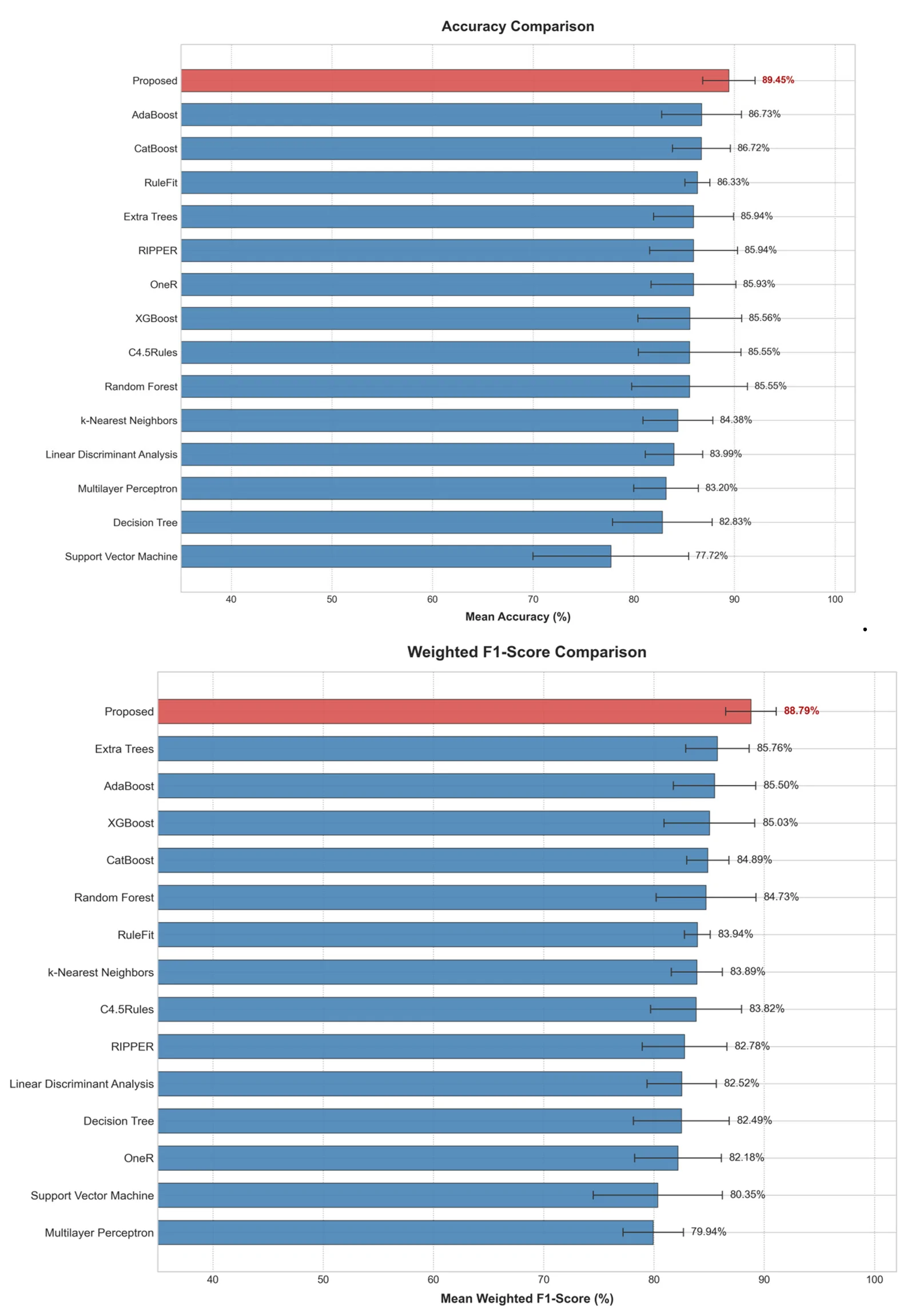
Co. arison of mean Accuracy and mean Weighted F1-score obtained by the proposed PSO-XGBoost with Partial SMOTE method and the baseline classification methods. Error bars indicate the variability across the five cross-validation folds.

The classification performance of the proposed PSO-XGBoost with Partial SMOTE method was compared with a diverse set of conventional machine learning, ensemble, and rule-based classification methods. All methods were evaluated under the same stratified five-fold cross-validation framework described in Section 3.2. The comparative results in terms of mean Accuracy and mean Weighted F1-score are presented in Figure 2. Error bars represent the variability observed across the five cross-validation folds.

As shown in Figure 2, the proposed PSO-XGBoost with Partial SMOTE method achieved the highest mean Accuracy among all evaluated methods, reaching 89.45%. Among the baseline methods, the highest mean Accuracy was obtained by AdaBoost (86.73%), followed by CatBoost (86.72%), RuleFit (86.33%), Extra Trees (85.94%), and RIPPER (85.94%). Conventional XGBoost achieved a mean Accuracy of 85.56%. Thus, the proposed method achieved an absolute improvement of 2.73 percentage points in mean Accuracy compared with the strongest baseline method, AdaBoost. A similar trend was observed for the mean Weighted F1-score, as shown in Figure 2. The proposed method achieved the highest mean Weighted F1-score of 88.79%. Among the baseline methods, Extra Trees achieved the highest mean Weighted F1-score of 85.76%, followed by AdaBoost (85.50%), XGBoost (85.03%), and CatBoost (84.89%). Accordingly, the proposed approach improved the mean Weighted F1-score by 3.03 percentage points compared with the strongest baseline result, obtained by Extra Trees.

The improvement in Weighted F1-score is particularly relevant because the dataset exhibits a considerable class imbalance, with substantially fewer observations belonging to the M class. While Accuracy reflects the overall proportion of correctly classified observations, Weighted F1-score incorporates class-specific precision and recall while accounting for class frequencies. The proposed method achieved the highest mean values for both Accuracy and Weighted F1-score among all evaluated methods. These findings indicate that the integration of Partial SMOTE with PSO-based XGBoost optimization provides a consistent performance advantage over the baseline classifiers evaluated under the same cross-validation framework. Partial SMOTE increases the representation of minority classes in a controlled manner, while PSO identifies an effective XGBoost hyperparameter configuration. Their combined use resulted in higher mean Accuracy and Weighted F1-score than those obtained by the individual baseline classifiers.

### 4.2. Detailed Performance Analysis of the Proposed Method

**Figure 3:**
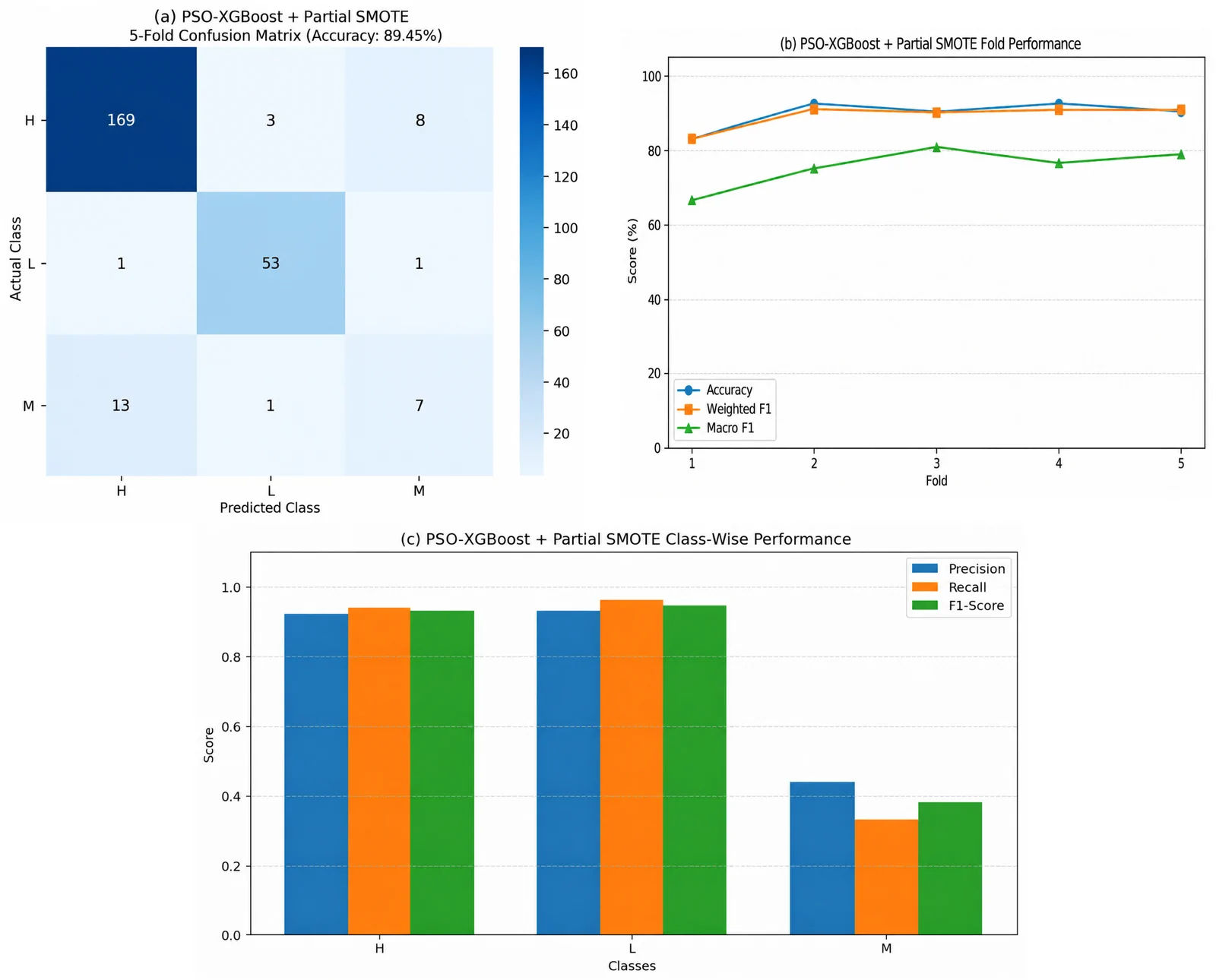
Detailed performance analysis of the proposed PSO-XGBoost with Partial SMOTE method: (**a**) aggregated confusion matrix based on out-of-fold predictions, (**b**) fold-wise Accuracy and Weighted F1-score, and (**c**) class-wise Precision, Recall, and F1-score.

The performance of the proposed PSO-XGBoost with Partial SMOTE method was further examined using the aggregated confusion matrix, fold-wise performance measures, and class-specific evaluation metrics, as presented in Figure 3.

Figure 3a presents the confusion matrix obtained by combining the out-of-fold predictions from the stratified five-fold cross-validation procedure. The proposed method correctly classified 169 of the 180 H observations and 53 of the 55 L observations. For class H, three observations were misclassified as L and eight as M. Class L showed particularly strong classification performance, with only one observation misclassified as H and one as M. The main classification difficulty was observed for class M. Of the 21 actual M observations, seven were correctly identified, while 13 were misclassified as H and one as L. Overall, 229 of the 256 observations were correctly classified, corresponding to an overall out-of-fold accuracy of 89.45%.

The fold-wise results shown in Figure 3b provide further insight into the stability of the proposed method across the five validation folds. Accuracy ranged from approximately 83% to 92%, while the Weighted F1-score varied within a similar range of approximately 83% to 91%. The highest Accuracy values were observed in the second and fourth folds, whereas the first fold produced the lowest value. The Macro F1-score showed greater variation, ranging from approximately 66% to 81%. The third fold achieved the highest Macro F1-score, while the first fold produced the lowest value. Despite this variation in Macro F1, the results across the remaining folds were relatively stable, indicating that the proposed method maintained consistent overall predictive performance. The difference between the overall and macro-averaged metrics primarily reflects the greater difficulty of correctly identifying the minority M class.

The class-specific Precision, Recall, and F1-score values are illustrated in Figure 3c. Classes H and L exhibited strong classification performance. For class H, Precision, Recall, and F1-score were approximately 92.35%, 93.89%, and 93.11%, respectively. Class L achieved a Precision of approximately 92.98%, Recall of 96.36%, and F1-score of 94.64%. These results indicate that the proposed method was highly effective in identifying observations belonging to the H and L classes.

In contrast, class M remained the most challenging class. The proposed method achieved a Precision of approximately 43.75%, Recall of 33.33%, and F1-score of 37.84% for this class. The Recall value indicates that 14 of the 21 actual M observations were not correctly identified, with most of these observations, 13 cases, being classified as H. The relatively low Precision and Recall values indicate that the discrimination of the M class remains more difficult than that of the H and L classes. This result should be interpreted in light of the considerable class imbalance in the original dataset, in which class M contains only 21 observations, compared with 55 observations for class L and 180 observations for class H.

Taken together, Figure 3 demonstrates that the proposed method achieved strong overall classification performance, particularly for classes H and L, while the identification of class M remained more challenging. The concentration of errors in the M class indicates that minority-class discrimination remains an important limitation despite the use of Partial SMOTE. Nevertheless, seven of the 21 M-class observations were correctly identified, corresponding to a Recall of 33.33%. Because Partial SMOTE was applied only to the training partitions within each cross-validation fold, the validation observations retained their original class distributions. Therefore, the reported out-of-fold results reflect model performance on non-oversampled observations and provide a more realistic assessment of generalization performance.

To further examine the interpretability of the proposed PSO-XGBoost with Partial SMOTE model, representative IF–THEN rules were extracted from the optimized XGBoost ensemble. As described in Section 3.3, each rule corresponds to a root-to-leaf decision path and expresses the associated temporal and meteorological conditions using bounded intervals. To illustrate the decision patterns learned for the three abundance classes, three representative rules were selected for each of the H, L, and M classes. The resulting rules and their corresponding rule-level accuracy values are presented in Table 2.

The representative rules for classes H and L exhibited high rule-level accuracy. Both selected H rules and both selected L rules achieved an accuracy of 1.00. For class M, one representative rule achieved an accuracy of 1.00, whereas the second achieved an accuracy of 0.62. However, the corresponding support and coverage values show that rule accuracy should be interpreted together with the number of observations represented by each rule. For example, the first M rule achieved an accuracy of 1.00 but covered only two observations, corresponding to 0.78% of the complete dataset, whereas the second M rule covered eight observations with an accuracy of 0.62. Rule-level accuracy was calculated over the complete original dataset by identifying all observations satisfying the conditions of each rule and determining the proportion belonging to the class predicted by that rule. Support represents the number of observations satisfying a rule, while coverage represents the proportion of the complete dataset represented by those observations.

The extracted rules also provide interpretable combinations of temporal and meteorological conditions associated with different *T. hirta* abundance levels. The H-class rules were mainly associated with early-season periods, specific DayOfYear intervals, temperature conditions, and high humidity. The L-class rules were primarily characterized by later DayOfYear intervals and atmospheric pressure conditions. The M-class rules involved combinations of minimum temperature, atmospheric pressure, wind speed, maximum temperature, and restricted DayOfYear intervals. These patterns suggest that abundance levels are associated with the joint influence of temporal and meteorological variables rather than with a single predictor. The temporal patterns identified for the H class are biologically plausible because adult *T. hirta* activity is known to coincide with the flowering period of fruit trees and to vary with environmental conditions such as temperature [2,3,4,5]. Nevertheless, the numerical intervals identified by the model should be interpreted as study-specific, data-driven associations rather than universal biological or economic thresholds.

An important characteristic of the extracted rules is that the predictor conditions are expressed using bounded intervals. When an XGBoost decision path provided only one boundary for a predictor, the missing boundary was completed using the minimum or maximum value of that predictor in the original dataset. This representation allows each condition to be expressed consistently in the form lower bound ≤ predictor ≤ upper bound, thereby providing a clearer description of the decision regions learned by the model and facilitating interpretation of the extracted rules.

It should also be emphasized that the rule-level accuracy, support, and coverage values reported in Table 2 are descriptive measures of the extracted rules and should not be interpreted as the overall predictive performance of the proposed classifier. The generalization performance of the proposed method was evaluated separately using stratified cross-validation and the additional robustness analyses reported in the Results section. Accordingly, Table 2 is intended to demonstrate the interpretability, consistency, and representativeness of selected decision rules, whereas the cross-validation results provide the appropriate assessment of predictive generalization.

### 4.3. Comparison with Alternative Hyperparameter Optimization Methods

To evaluate the effectiveness of PSO as a hyperparameter optimization strategy, the proposed approach was compared with Bayesian Optimization and Random Search under the same XGBoost + Partial-SMOTE framework. The comparison results are presented in Table 3.

As shown in Table 3, the proposed approach achieved the best overall performance among the three hyperparameter optimization methods. The proposed method achieved the highest mean Accuracy of 89.45% and the highest mean Weighted F1-score of 88.79%. Bayesian Optimization and Random Search both achieved a mean Accuracy of 88.69%, with Weighted F1-scores of 88.11% and 88.49%, respectively. The proposed method also achieved the highest Macro F1-score of 75.09%, followed closely by Random Search at 75.06%, while Bayesian Optimization achieved a Macro F1-score of 73.57%. Overall, these results indicate that PSO provided the most favorable overall performance across the evaluated metrics and was competitive with commonly used alternative hyperparameter optimization strategies.

### 4.4. Partial-SMOTE Ratio Ablation Analysis

To examine the effect of the oversampling ratios used in Partial-SMOTE, an ablation analysis was conducted using different target ratios for classes L and M. During these experiments, the XGBoost hyperparameters were kept fixed so that the effect of the oversampling ratios could be evaluated independently. The analysis also included a no-SMOTE baseline and the original L = 0.60, M = 0.45 configuration. The representative results of the ablation analysis are presented in Table 4.

As shown in Table 4, the choice of Partial-SMOTE ratios had a clear effect on classification performance. The no-SMOTE baseline achieved a mean Accuracy of 87.50%, a Weighted F1-score of 83.74%, and a Macro F1-score of 59.91%. Among the evaluated Partial-SMOTE configurations, the L = 0.80 and M = 0.40 setting achieved the best overall performance, with a mean Accuracy of 89.45%, a mean Weighted F1-score of 88.79%, and a mean Macro F1-score of 75.76%. These results indicate that controlled class-specific oversampling can improve both overall and class-sensitive predictive performance without requiring complete class balancing. Based on this sensitivity analysis, L = 0.80 and M = 0.40 were selected as the final Partial-SMOTE ratios used in the proposed method.

Compared with the original configuration, the L = 0.80, M = 0.40 setting increased Accuracy by 1.57 percentage points, Weighted F1-score by 1.88 percentage points, and Macro F1-score by 5.00 percentage points. The larger improvement in Macro F1 indicates that the revised oversampling ratios provided a more balanced classification performance across the three classes. Furthermore, increasing the minority-class ratios beyond this level did not consistently improve performance, indicating that more aggressive oversampling was not necessarily beneficial. Based on these results, L = 0.80 and M = 0.40 were selected as the Partial-SMOTE ratios for the final model configuration.

### 4.5. Ablation Study of the Proposed Method

To investigate the contribution of the individual components of the proposed method, an ablation analysis was conducted by progressively modifying the baseline XGBoost framework. As presented in Table 5, four configurations were considered: conventional XGBoost, PSO-optimized XGBoost, PSO-XGBoost combined with Full SMOTE, and the proposed PSO-XGBoost with Partial SMOTE approach.

The application of Full SMOTE produced a different performance pattern. Compared with PSO-XGBoost without oversampling, the mean Accuracy decreased slightly from 87.90% to 87.11%, whereas the mean Weighted F1-score increased from 86.05% to 88.07%. As shown in Table 5, the proposed PSO-XGBoost with Partial SMOTE approach achieved the highest mean Accuracy and Weighted F1-score among all evaluated configurations. The method obtained a mean Accuracy of 89.45%, corresponding to improvements of 3.90 percentage points over conventional XGBoost (85.56%), 1.55 percentage points over PSO-XGBoost without oversampling (87.90%), and 2.34 percentage points over PSO-XGBoost with Full SMOTE (87.11%). Similarly, the proposed method achieved the highest mean Weighted F1-score of 88.79%, outperforming conventional XGBoost (85.03%), PSO-XGBoost (86.05%), and PSO-XGBoost with Full SMOTE (88.07%) by 3.76, 2.74, and 0.72 percentage points, respectively.

These results indicate that the selected Partial SMOTE strategy provided the most favorable balance between overall classification accuracy and F1-based predictive performance among the evaluated configurations. Overall, the ablation results demonstrate that the components of the proposed framework affected the two evaluation metrics differently. PSO-based optimization improved both Accuracy and Weighted F1-score relative to conventional XGBoost. Full SMOTE increased the Weighted F1-score compared with PSO-XGBoost without oversampling but resulted in a slight reduction in Accuracy. In contrast, the proposed Partial SMOTE configuration achieved both the highest mean Accuracy of 89.45% and the highest mean Weighted F1-score of 88.79%. These findings suggest that Partial SMOTE provides a controlled alternative to complete class balancing and achieved the best overall performance among the evaluated configurations.

### 4.6. Repeated Stratified Cross-Validation and Statistical Reliability

To further evaluate the robustness and stability of the comparative results across different data partitions, the four principal XGBoost-based methods were assessed using 10-times repeated stratified five-fold cross-validation, and the corresponding performance results are presented in Table 6.

As shown in Table 6, the proposed PSO-XGBoost with Partial SMOTE approach achieved the highest mean performance across all three evaluation metrics. The method obtained an Accuracy of 87.51 ± 3.75%, a Weighted F1-score of 86.94 ± 3.71%, and a Macro F1-score of 71.60 ± 7.40%. Compared with conventional XGBoost, the proposed approach improved mean Accuracy by 2.49 percentage points and Weighted F1-score by 2.42 percentage points. It also outperformed PSO-XGBoost without Partial SMOTE by 1.05 percentage points in Accuracy and 1.50 percentage points in Weighted F1-score. The larger improvement observed in Macro F1 indicates that Partial SMOTE particularly contributed to more balanced predictive performance across the imbalanced classes. Overall, the repeated cross-validation results demonstrate that the advantage of the proposed method was consistently maintained across different stratified data partitions rather than being dependent on a single five-fold split.

### 4.7. Statistical Significance Analysis of Repeated Cross-Validation Results

Following the repeated stratified cross-validation results presented in Table 6, statistical significance analyses were conducted to determine whether the observed performance differences among the evaluated XGBoost-based methods were statistically meaningful. First, the Friedman test was applied to the repeat-level Accuracy, Weighted F1-score, and Macro F1-score values to assess the overall differences among the four methods. The corresponding results are presented in Table 7.

As shown in Table 7, the Friedman test revealed statistically significant differences among the four evaluated methods for all three performance metrics. Significant differences were observed for Accuracy (χ^2^ = 25.56, *p* = 1.18 × 10^−5^), Weighted F1-score (χ^2^ = 25.92, *p* = 9.91 × 10^−6^), and Macro F1-score (χ^2^ = 24.12, *p* = 2.36 × 10^−5^). Since all *p*-values were substantially below the significance threshold of 0.05, the null hypothesis that the four methods exhibited equivalent performance was rejected for each metric.

Following the significant overall differences identified by the Friedman test, post-hoc pairwise comparisons were performed using the two-sided Wilcoxon signed-rank test. To control the family-wise error rate arising from multiple comparisons, the resulting *p*-values were adjusted using the Holm procedure. The comparisons between the proposed PSO-XGBoost with Partial SMOTE method and the competing approaches are presented in Table 8.

As shown in Table 8, the proposed PSO-XGBoost with Partial SMOTE approach exhibited statistically significant improvements over all three competing methods. For Accuracy, significant differences were obtained against XGBoost (adjusted *p* = 0.00586), class-weighted XGBoost (adjusted *p* = 0.00586), and PSO-XGBoost (adjusted *p* = 0.02734). Similarly, the Weighted F1-score comparisons were significant against XGBoost, class-weighted XGBoost, and PSO-XGBoost, with Holm-adjusted *p*-values of 0.00586 in all three comparisons. The same statistically significant pattern was observed for Macro F1-score. These findings support the repeated cross-validation results in Table 6 and indicate that the performance gains achieved by the proposed method are not attributable solely to variations in the data partitioning.

### 4.8. Additional Nested Cross-Validation Analysis

To further assess the generalization performance of the proposed method under a stricter evaluation design, an additional nested cross-validation experiment was conducted. In this analysis, the outer five-fold cross-validation loop was used exclusively for final performance evaluation, while PSO-based hyperparameter optimization was performed within each outer training partition using an inner five-fold stratified cross-validation procedure. Thus, the outer test folds were completely isolated from the hyperparameter selection process. Under this nested evaluation, the proposed method achieved a mean Accuracy of 86.33 ± 4.78%, a mean Weighted F1-score of 85.30 ± 3.16%, and a mean Macro F1-score of 68.26 ± 3.90%. These results provide an additional, more conservative assessment of generalization performance and confirm that the proposed framework retains competitive predictive capability when hyperparameter selection is strictly separated from final testing.

## 5. Discussion

The results of this study demonstrate that PSO-based hyperparameter optimization combined with controlled oversampling can improve the classification of *T. hirta* abundance levels from temporal and meteorological variables. The proposed PSO-XGBoost with Partial SMOTE method achieved a mean Accuracy of 89.45% and a mean Weighted F1-score of 88.79%, outperforming the conventional XGBoost and the other evaluated configurations. The ablation analysis showed that PSO optimization increased Accuracy from 85.56% to 87.90% and Weighted F1-score from 85.03% to 86.05%, while Partial SMOTE further improved overall performance. In addition, the class-weighted XGBoost baseline did not provide an advantage over the conventional XGBoost model, indicating that the controlled oversampling strategy was more effective under the present experimental conditions.

The Partial-SMOTE ratio analysis further showed that the degree of oversampling affected model performance. Among the tested configurations, the L = 0.80 and M = 0.40 setting achieved the best overall results, with an Accuracy of 89.45%, Weighted F1-score of 88.79%, and Macro F1-score of 75.76%. This result indicates that complete class balancing is not necessarily optimal and that controlled minority-class oversampling can provide a better balance between overall accuracy and class-sensitive performance.

The robustness of the proposed method was also supported by 10-times repeated stratified five-fold cross-validation. The proposed approach achieved an Accuracy of 87.51 ± 3.75%, Weighted F1-score of 86.94 ± 3.71%, and Macro F1-score of 71.60 ± 7.40%, with the highest mean performance among the evaluated XGBoost-based methods. Furthermore, Friedman and post-hoc Wilcoxon tests with Holm correction showed that the observed improvements were statistically significant, indicating that the performance advantage was not dependent on a single data partition.

Another important contribution of the framework is its interpretability through human-readable IF–THEN rules. The extracted rules describe combinations of seasonal and meteorological conditions associated with different abundance classes. In particular, several high-abundance rules were associated with early-season periods and moderate temperature ranges, which is biologically plausible because adult *T. hirta* activity coincides with the flowering period of fruit trees and is influenced by environmental conditions such as temperature [2,3,4,5]. However, these intervals should be interpreted as study-specific, data-driven associations rather than universal biological thresholds.

From a practical perspective, the model is intended to support pest-abundance prediction, monitoring, and pest-management decision-making rather than directly prescribe pest-control treatments. When a high-abundance condition is predicted, orchard managers could increase blue-trap inspection and field-scouting frequency, focus monitoring efforts on higher-risk periods, and allocate management resources more efficiently. Since *T. hirta* activity overlaps with flowering and pollinator activity, model predictions should be combined with actual trap catches, field observations, crop phenology, and local pest-management recommendations before any intervention decision is made.

Several limitations should also be considered. The dataset contained only 256 observations and remained substantially imbalanced, particularly for the M class, which contained only 21 observations and showed lower classification performance. Despite the use of Partial SMOTE, the Medium class remained difficult to identify, with a recall of 19.05% under nested cross-validation. This indicates that the proposed method mitigates, but does not fully resolve, the class-imbalance problem, particularly for the smallest minority class. In addition, the data were obtained from a limited observational setting, and external validation across different orchards, seasons, and climatic regions has not yet been performed. Because the main cross-validation experiments used shuffled stratified partitions, temporally related observations may have appeared in both training and validation folds. Therefore, temporal, year-based, or grouped validation should be considered in future studies using larger multi-season datasets to further assess temporal generalizability. Partial SMOTE also introduces synthetic training observations, which may influence the learned decision boundaries. Finally, the abundance categories used in this study are study-specific operational classes and should not be interpreted as economic injury or pesticide-application thresholds.

Future studies should validate the framework using larger and more geographically diverse datasets, increase the representation of the minority M class, and examine the stability of the extracted rules across seasons and orchards. Integration with near-real-time meteorological and trap-monitoring data may also support the development of practical orchard-level early-warning systems.

## 6. Conclusions

This study proposed a PSO-XGBoost with Partial SMOTE framework for classifying Low, Medium, and High abundance levels of *T. hirta* derived from blue basin trap captures in almond orchards. By integrating temporal and meteorological variables, controlled oversampling, bio-inspired PSO-based hyperparameter optimization, XGBoost classification, and interpretable rule extraction, the proposed framework was designed to improve both predictive performance and model transparency. From a biomimetic perspective, the framework transfers principles of collective search, information sharing, and adaptive behavior observed in natural swarms to the optimization of XGBoost hyperparameters.

The experimental results showed that the proposed PSO-XGBoost with Partial SMOTE method achieved a mean Accuracy of 89.45% and a mean Weighted F1-score of 88.79%, outperforming the evaluated baseline and alternative XGBoost-based configurations. The ablation analysis demonstrated that PSO-based hyperparameter optimization improved conventional XGBoost, increasing mean Accuracy from 85.56% to 87.90% and mean Weighted F1-score from 85.03% to 86.05%. The Partial-SMOTE ratio analysis further showed that the degree of oversampling influenced predictive performance, with the L = 0.80 and M = 0.40 configuration achieving the most favorable overall balance, including an Accuracy of 89.45%, Weighted F1-score of 88.79%, and Macro F1-score of 75.76%. These findings indicate that controlled oversampling can provide a more effective alternative to complete class balancing under the investigated class-imbalanced conditions.

The robustness of the proposed method was further supported by 10-times repeated stratified five-fold cross-validation, in which it achieved an Accuracy of 87.51 ± 3.75%, a Weighted F1-score of 86.94 ± 3.71%, and a Macro F1-score of 71.60 ± 7.40%. Statistical analyses based on the Friedman test and post-hoc Wilcoxon signed-rank tests with Holm correction also confirmed that the performance improvements over the competing XGBoost-based methods were statistically significant.

The extracted IF–THEN rules further enhanced interpretability by expressing model decisions through bounded temporal and meteorological conditions. These rules provide human-readable descriptions of the environmental patterns associated with different abundance levels and complement the predictive capability of the optimized XGBoost model. However, the identified rule intervals should be interpreted as study-specific, data-driven associations rather than universal biological or intervention thresholds.

Despite the promising results, several limitations remain. The dataset contained only 256 valid observations and remained substantially imbalanced, particularly for the minority M class. In addition, the data were obtained from a limited observational setting, and external validation across different orchards, seasons, geographical regions, and climatic conditions has not yet been performed. Future studies should therefore evaluate the framework using larger and more diverse datasets, investigate adaptive or optimization-based oversampling strategies, and assess the stability of the extracted rules across independent environments. Overall, the results indicate that combining blue-trap monitoring, controlled oversampling, biomimetic PSO-based optimization, and interpretable rule extraction provides a promising framework for data-driven *T. hirta* abundance prediction, monitoring, and orchard-level pest-management decision support under class-imbalanced conditions.

## Figures and Tables

**Table 1:** Summary of the variables used in the dataset.

Variable	Role	Description
Year	Predictor	Year in which the observation was recorded
Month	Predictor	Month in which the observation was recorded
DayOfYear	Predictor	Sequential day of the year
Max. Temperature	Predictor	Maximum daily temperature
Min. Temperature	Predictor	Minimum daily temperature
Mean Temperature	Predictor	Mean daily temperature
Humidity	Predictor	Relative humidity
Precipitation	Predictor	Precipitation amount
Wind Speed	Predictor	Wind speed
Wind Angle	Predictor	Wind direction expressed as an angle
Atmospheric Pressure	Predictor	Atmospheric pressure
PestCount	Excluded	Observed pest abundance; used to derive the target class

**Table 2:** Representative IF–THEN classification rules extracted from the proposed PSO-XGBoost with Partial SMOTE method.

Rule	Class	Accuracy	Correct Cases	Support	Coverage (%)
IF 3.14 ≤ Month ≤ 4.00 AND 62.00 ≤ DayOfYear ≤ 105.07 AND 5.20 ≤ Max. Temperature ≤ 16.20 AND −0.60≤ Min. Temperature ≤ 7.33	H	1.00	34	34	13.28
IF 88.21 ≤ DayOfYear ≤ 110.03 AND 81.00 ≤ Humidity ≤ 90.00	H	1.00	20	20	7.81
IF 111.09 ≤ DayOfYear ≤ 118.00	L	1.00	26	26	10.16
IF 91.23 ≤ DayOfYear ≤ 108.29 AND 1012.80 ≤ Pressure ≤ 1021.10	L	1.00	18	18	7.03
IF 5.24 ≤ Min. Temperature ≤ 12.70 AND 1017.33 ≤ Pressure ≤ 1021.10	M	1.00	2	2	0.78
IF 6.90 ≤ Wind Speed ≤ 35.50 AND 16.95 ≤ Max. Temperature ≤ 25.50 AND 88.00 ≤ DayOfYear ≤ 91.23	M	0.62	5	8	3.13

**Table 3:** Comparison of PSO, Bayesian Optimization, and Random Search for XGBoost hyperparameter optimization with Partial-SMOTE.

Method	Accuracy (%)	Weighted F1 (%)	Macro F1 (%)
Proposed	89.45 ± 2.60	88.79 ± 2.29	75.09 ± 3.60
Bayesian Optimization	88.69 ± 4.48	88.11 ± 3.60	73.57 ± 6.07
Random Search	88.69 ± 3.30	88.49 ± 2.59	75.06 ± 3.54

**Table 4:** Ablation analysis of Partial-SMOTE oversampling ratios.

Oversampling Strategy	Accuracy (%)	Weighted F1 (%)	Macro F1 (%)
No SMOTE	87.50 ± 2.64	83.74 ± 2.62	59.91 ± 2.60
L = 0.60, M = 0.45	87.91 ± 4.46	86.99 ± 4.28	70.76 ± 6.52
L = 0.70, M = 0.50	88.69 ± 2.79	87.92 ± 1.38	72.31 ± 4.25
L = 0.40, M = 0.40	89.09 ± 3.73	88.04 ± 4.15	73.55 ± 7.46
L = 0.80, M = 0.40	89.45 ± 2.60	88.79 ± 2.29	75.76 ± 4.76

**Table 5:** Ablation analysis of the components of the proposed PSO-XGBoost with Partial SMOTE method.

Method	Accuracy (%)	Weighted F1 (%)
XGBoost	85.56	85.03
Class-Weighted XGBoost	85.17 ± 5.17	84.94 ± 4.62
PSO-XGBoost	87.90 ± 3.75	86.05 ± 3.38
PSO-XGBoost + Full SMOTE	87.11 ± 4.39	88.07 ± 3.39
PSO-XGBoost + Partial SMOTE	89.45 ± 2.60	88.79 ± 2.29

**Table 6:** Performance comparison under 10-times repeated stratified five-fold cross-validation.

Method	Accuracy (%)	Weighted F1 (%)	Macro F1 (%)
XGBoost	85.02 ± 4.39	84.52 ± 3.46	66.97 ± 5.61
Class-Weighted XGBoost	84.82 ± 4.16	84.51 ± 3.32	67.19 ± 5.62
PSO-XGBoost	86.46 ± 3.63	85.44 ± 3.23	68.19 ± 5.92
PSO-XGBoost + Partial SMOTE	87.51 ± 3.75	86.94 ± 3.71	71.60 ± 7.40

**Table 7:** Friedman test results for the performance metrics obtained from repeated stratified cross-validation.

Metric	Friedman χ^2^	*p*-Value
Accuracy	25.56	1.18 × 10^−5^
Weighted F1	25.92	9.91 × 10^−6^
Macro F1	24.12	2.36 × 10^−5^

**Table 8:** Post-hoc Wilcoxon signed-rank test results with Holm correction.

Metric	Pairwise Comparison	Wilcoxon W	Holm-Adjusted *p*-Value
Accuracy	Proposed vs. XGBoost	0	0.00586
	Proposed vs. Class-Weighted XGBoost	0	0.00586
	Proposed vs. PSO-XGBoost	6	0.02734
Weighted F1	Proposed vs. XGBoost	0	0.00586
	Proposed vs. Class-Weighted XGBoost	0	0.00586
	Proposed vs. PSO-XGBoost	2	0.00586
Macro F1	Proposed vs. XGBoost	0	0.00586
	Proposed vs. Class-Weighted XGBoost	0	0.00586
	Proposed vs. PSO-XGBoost	2	0.00586

## Data Availability

The dataset supporting the findings of this study is provided as Supplementary Material accompanying this article.

## Supplementary Materials

The following supporting information can be downloaded at https://www.mdpi.com/article/10.3390/biomimetics11100738/s1, Dataset S1: Dataset used for the classification of *Tropinota hirta* abundance levels.

## Abbreviations

The following abbreviations are used in this manuscript:

PSO	Particle Swarm Optimization
SMOTE	Synthetic Minority Oversampling Technique
XGBoost	Extreme Gradient Boosting
RF	Random Forest
ACO	Ant Colony Optimization
SHAP	SHapley Additive exPlanations
SVM	Support Vector Machine
LDA	Linear Discriminant Analysis
CNN	Convolutional Neural Network
LSTM	Long Short-Term Memory
